# Machine learning for distinguishing saudi children with and without autism via eye-tracking data

**DOI:** 10.1186/s13034-023-00662-3

**Published:** 2023-09-30

**Authors:** Hana Alarifi, Hesham Aldhalaan, Nouchine Hadjikhani, Jakob Åsberg Johnels, Jhan Alarifi, Guido Ascenso, Reem Alabdulaziz

**Affiliations:** 1 Autism Center, King Faisal Specialists Hospital and Research Center, Riyadh, Kingdom of Saudi Arabia; 2 Neurolimbic Research, Harvard/MGH Martinos Center for Biomedical Imaging, Charlestown, MA USA; 3 Gillberg Neuropsychiatry Centre, University of Gothenburg, Gothenburg, Sweden; 4 Section of Speech and Language Pathology, Institute of Neuroscience and Physiology, University of Gothenburg, Gothenburg, Sweden; 5 Department of Electronics, Information, and Bioengineering, Politecnico di Milano, Milan, Italy

**Keywords:** Autism spectrum disorder, Eye-tracking, Face Processing, Prediction, Screening

## Abstract

**Background:**

Despite the prevalence of Autism Spectrum Disorder (ASD) globally, there’s a knowledge gap pertaining to autism in Arabic nations. Recognizing the need for validated biomarkers for ASD, our study leverages eye-tracking technology to understand gaze patterns associated with ASD, focusing on joint attention (JA) and atypical gaze patterns during face perception. While previous studies typically evaluate a single eye-tracking metric, our research combines multiple metrics to capture the multidimensional nature of autism, focusing on dwell times on eyes, left facial side, and joint attention.

**Methods:**

We recorded data from 104 participants (41 neurotypical, mean age: 8.21 ± 4.12 years; 63 with ASD, mean age 8 ± 3.89 years). The data collection consisted of a series of visual stimuli of cartoon faces of humans and animals, presented to the participants in a controlled environment. During each stimulus, the eye movements of the participants were recorded and analyzed, extracting metrics such as time to first fixation and dwell time. We then used these data to train a number of machine learning classification algorithms, to determine if these biomarkers can be used to diagnose ASD.

**Results:**

We found no significant difference in eye-dwell time between autistic and control groups on human or animal eyes. However, autistic individuals focused less on the left side of both human and animal faces, indicating reduced left visual field (LVF) bias. They also showed slower response times and shorter dwell times on congruent objects during joint attention (JA) tasks, indicating diminished reflexive joint attention. No significant difference was found in time spent on incongruent objects during JA tasks. These results suggest potential eye-tracking biomarkers for autism. The best-performing algorithm was the random forest one, which achieved accuracy = 0.76 ± 0.08, precision = 0.78 ± 0.13, recall = 0.84 ± 0.07, and F1 = 0.80 ± 0.09.

**Conclusions:**

Although the autism group displayed notable differences in reflexive joint attention and left visual field bias, the dwell time on eyes was not significantly different. Nevertheless, the machine algorithm model trained on these data proved effective at diagnosing ASD, showing the potential of these biomarkers. Our study shows promising results and opens up potential for further exploration in this under-researched geographical context.

## Background

Autism spectrum disorder (ASD) is a neurodevelopmental condition characterized by variations in social and communicative development, sensory changes, and limitations in behaviors and interests. Most autism research stems from Western nations, with relatively limited knowledge on the features, appropriate assessment tools, and individual differences of individuals with autism in Arabic countries. A large-scale study in Qatar recently revealed an autism prevalence of 1.14% there, in alignment with findings from international studies [1]. In clinical practice, ASD diagnoses are based on an individual’s symptoms and developmental history. However, in group studies, autism has been linked to dysfunction in various neurocognitive systems [2]. For instance, increasing evidence suggests that ASD is associated with impairments in joint attention (JA) [4] and atypical gaze patterns when viewing social information such as faces [5]. Eye-tracking technology, being non-invasive and user-friendly, has the potential to provide biomarkers for ASD when used alongside stimuli and experimental designs reflecting autism’s atypical behavioral profile [6].

Research consistently shows that people tend to focus on the central areas of faces, particularly the eyes, when looking at others’ faces [7]. However, eye-tracking studies on children with autism have demonstrated a reduced focus on the eye region [8], [9]. Therefore, our current study collected data on gaze duration at the eye region during face perception in Saudi children with and without autism. Eye-tracking technology has also revealed that individuals typically focus first and longer on the face’s left hemifield, displaying a “left visual field bias” (LVF) during face perception, which is linked to right hemispheric dominance for face processing [10]. This LVF bias is weaker or absent in toddlers [9] and adults [11], [12] with autism, suggesting a less specialized face-processing system. Moreover, Guillon et al. [9] found gaze differences in toddlers with autism when viewing dog faces and human cartoon faces. In this study, we examined LVF bias by analyzing total gaze duration on the left versus the right side of human and animal cartoon faces, and we also measured the time spent gazing on the eyes region.

Joint attention (JA) is an essential developmental precursor to social and language development in children. Typically developing infants begin to exhibit JA during the latter half of their first year [13], which involves responding to another person’s gaze direction by looking at the same objects and events. JA impairments are an early indicator of autism, and JA skills are linked to natural outcomes in autism, making them a promising intervention target [4]. JA is often assessed in autism diagnostic tools, such as the ADOS [14]. However, accurate JA coding can be challenging and time-consuming. In this study, we employed eye movement recordings [15], [16], [6] to objectively assess JA in terms of reflexive gaze following.

The complexity of autism diagnosis and the limited screening methods designed for screening in different regions with cultural and communication differences such as Saudi Arabia recently motivated researchers to explore objective measurements for autism screening. A recent study examining the usability of eye-tracking data as a screening system for autism tested for a specific group in Saudi Arabia found that the Support Vector Machine algorithm was useful to distinguish children with and without autism, with accuracy of 88.6%, specificity of 92.31%, and sensitivity 86.63% [6]. Another research at Najran University in Saudi Arabia investigated eye tracking-based diagnosis using machine learning by utilizing open datasets which includes over 500 images of scan paths for 29 individuals with ASD and 30 control group participants. The findings of this research recommend using hybrid artificial neural networks and support vector machine algorithms.

A recent systematic review of eye-tracking autism screening studies [21] explored 24 studies between 2015 and 2021 that used machine learning for autism classification, for a total population of 1396 individuals. The performance varied in the range of groups (accuracy = 81%, specificity = 79%, sensitivity = 84%). However, the variation of implementation methods reduces the reliability of the results, especially as none of the studies performed a thorough comparison between different methods, but just considered them in isolation. Moreover, none of the included studies were specific to this study’s target group, which includes differences in language and culture that may affect the perception of participants where group specific studies are encouraged to explore further in this domain [21].

The aim of this study was to compare the gaze patterns of Saudi children with and without autism on three face-processing metrics (gaze duration at the eye region, LVF bias, and JA) and investigate the potential of these metrics for autism diagnosis classification for this specific population, on which little to nothing is documented in the literature. Considering autism’s multidimensional nature, we hypothesized that combining multiple eye-tracking metrics could enhance autism identification accuracy. To test this, we compared groups on each metric and implemented several traditional machine learning (ML) models for diagnostic classification. The goal of the algorithms was to be able to correctly classify patients as having ASD or not based on their eye-tracking data. As such methods can operate in real time once trained, they would make powerful screening tools, shortening the currently long waiting lists for performing ASD screening tests with traditional methods.

## Methods

**Fig. 1:**
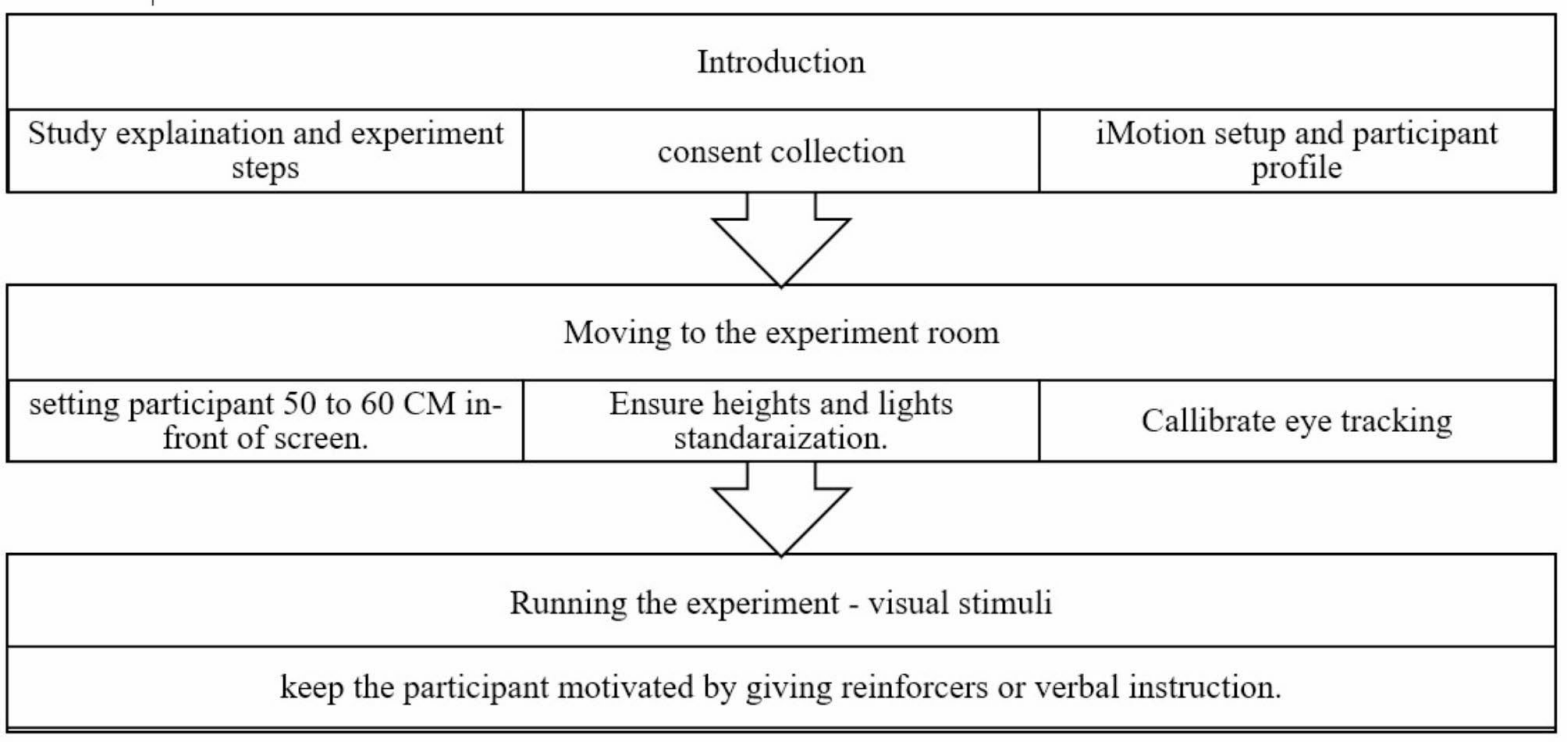
Experiment procedure steps

The experiment followed the steps reported in Fig. 1 and described in detail in the next sections. Ethical approval was obtained from King Faisal Specialist Hospital and Research Centre (RAC – 2,201,183). The experiments took place in the Human Behavior Lab of the hospital. Participants were welcomed to the lab and informed about the procedure upon arrival. Consent forms were collected with the permission of the participants’ guardians.

### Participants

A participant recruitment form was electronically disseminated through email and the hospital’s official social media channels to current patients at the center and those on the waiting list. A total of 130 participants were enrolled for this study. Of these, 28 were later excluded from the analysis due to inadequate calibration or because their measurements were invalid (e.g., they did not look at the screen during one or more visual stimuli). The final number of participants with satisfactory calibration and engagement rate was 104 (74 males, 30 females): 41 neurotypical (23 male, 18 female), 63 with ASD (51 male, 12 female). In the ASD group, the youngest participant was one year and ten months old, and the oldest was 22 years old; in the neurotypical group, the age range spanned from two to 17 years old. The mean age was 8 ± 3.89 years for the ASD group and 8.21 ± 4.12 years for neurotypical group. The two groups were comparable in terms of age (t = 0:26(72); p = 0:797; d = -0:05) and gender (χ^2^ = 19:36(107); p = 1). Consequently, we deemed our sample heterogeneous, accurately representing the population of clinic attendees in Saudi Arabia.

The Social Communication Questionnaire (SCQ) was collected for most participants in both groups (N = 94). A cut-off score of 14 was employed to identify the risk of further assessment for autism, which is one of the clinic’s initial screening processes. Confirmation of an ASD diagnosis was carried out at the clinic by a multidisciplinary team comprising five divisions: pediatric neurology, psychology, occupational therapy and sensory integration, speech and language pathology, and behavioral analysis and observation. In our ASD sample, 60% (N = 36) of participants also had their ASD diagnosis confirmed using ADOS. In all instances, DSM-5 criteria were applied.

### Setup

The testing room was specifically tailored for this experiment, with careful attention given to maintaining consistent lighting, ensuring a constant distance of 50 to 60 cm between the participant and the screen, and standardizing the height at which participants saw the screen by using an adjustable chair and footrest; the average height from the floor to the center of the screen was 114 cm.

**Fig. 2:**
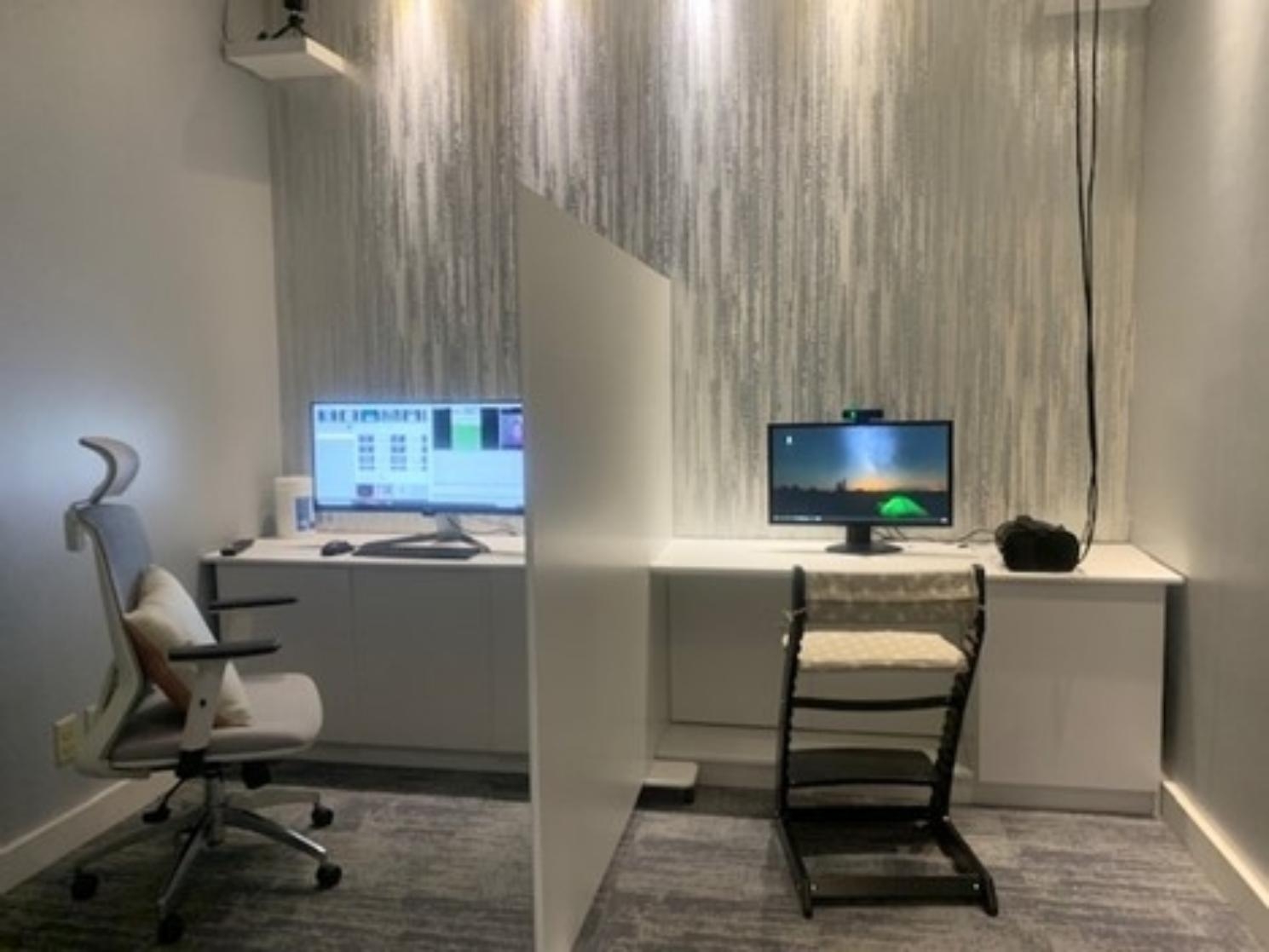
Experiment room setup

The experimental setup minimized distractions by having participants sit in front of the screen, separated from the control computer by a divider. Parents could observe their children from behind a one-way glass mirror positioned behind the participant. Some participants preferred to have a parent present and to sit on their lap. In such cases, the parent was asked to wear black sunglasses to prevent data contamination. In compliance with the ethical approval obtained for the study, one parent was always present during the experiment, either inside the room wearing black sunglasses or behind the one-way glass mirror. Figure 2 shows the experimental setup utilized for data collection.

### Data collection procedure

Visual stimuli were specifically designed for this study to investigate differences in visual behavior during social perception tests, including left visual field (LVF) bias, joint attention, and perception of human and animal cartoon faces. To examine LVF bias and eye gaze, four distinct child avatar faces were created. Each stimulus was displayed for a total of four seconds while participants’ gaze patterns were recorded. Moreover, we included images of four animal cartoon faces to explore potential similarities and differences in gaze patterns compared to human faces.

**Fig. 3:**
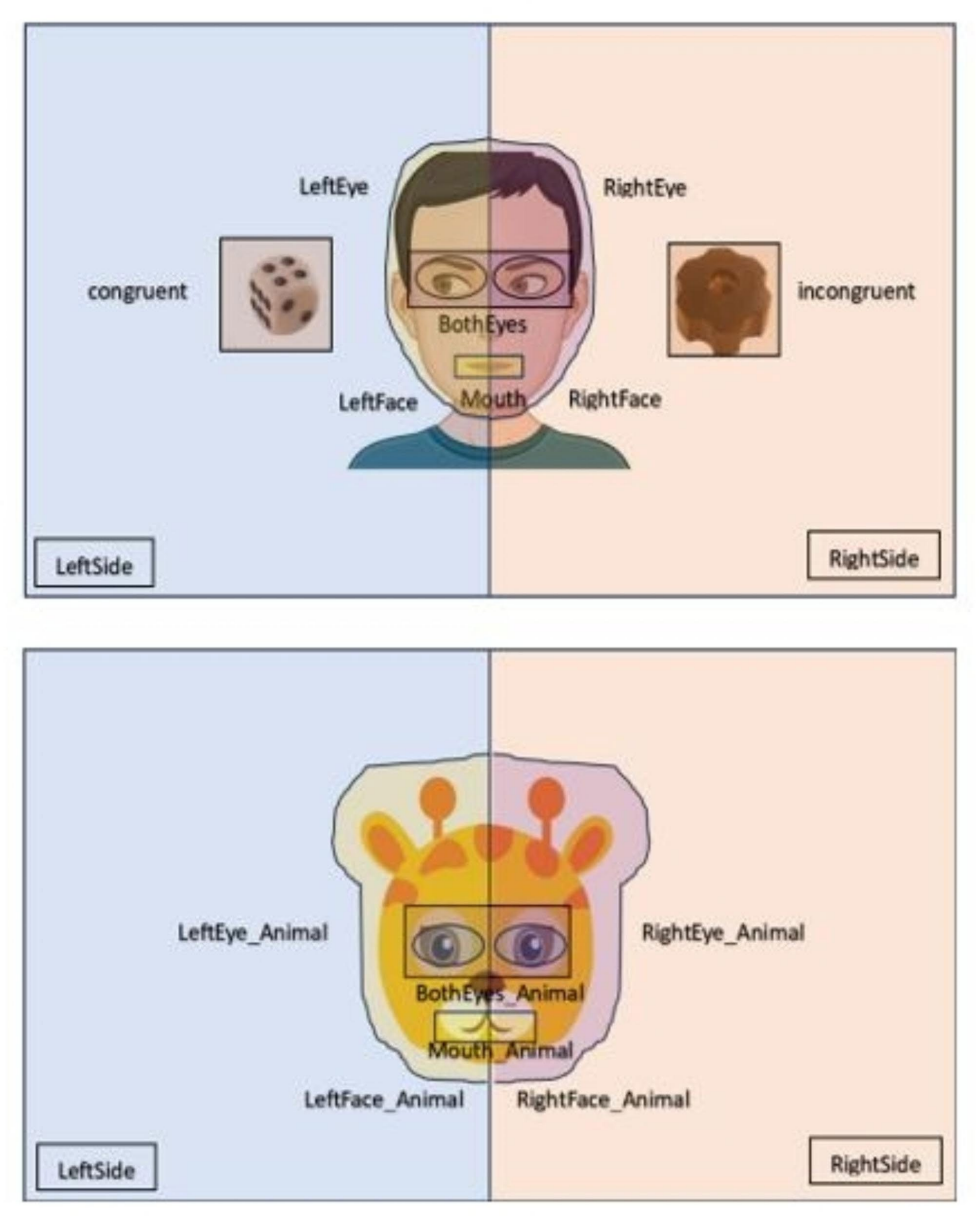
Examples of the visual stimuli and AOI for the JA task

To assess joint attention, we presented images of children avatars with their gaze directed towards one of two toys, arranged in a numerically balanced and pseudo-randomized fashion on either the left or the right side of the child avatar (Fig. 3). In total, four child avatar identities were shown, each appearing twice with the target toy on either the left or right side. The time taken to achieve the first fixation and the duration participants spent looking at the object gazed upon by the avatars were recorded as the primary outcome measures. It was anticipated that children with autism would be less inclined to direct their attention towards the object viewed by the avatars. Participants were simply instructed to “look at the screen” during the task, with no additional guidance provided. Additional metrics were examined during the experiment, using stimuli probing scene perception, visual disengagement, and pupillary reactions; these results will be reported in a separate paper. The experiments were conducted using iMotions software version 8.3 [17]. Visual stimuli were displayed on a 24-inch screen with a resolution of 1920 × 1080 pixels. Areas of interest (AOI) were designated for each image shown. A screen-based eye tracker by Tobii Pro Fusion, which captured gaze data at a maximum frequency of 120 Hz [18], was utilized for eye-tracking.

### Data pre-processing

Relevant areas of interest (AOIs) were drawn on each of the stimuli using the iMotions software. For condition one (Attention on Eyes), two AOIs were drawn on the eyes. For condition two (LVF Bias), AOIs were drawn on the left and right sides of the screen. For condition three (Joint Attention), AOIs were drawn for congruent and incongruent objects. From the iMotions AOI Metrics, ‘dwell time’ (defined below) was exported for each AOI per participant per stimuli. Further, ‘time to first fixation (TTFF)’ (defined below) was exported for condition three. Finally, mean values were calculated per group and t-tests were calculated to measure between-group differences on these metrics. All statistical analyses were done in RStudio. Outliers or participants with missing data (one to three per prediction) were removed.

In total, the dataset consisted of a binary label (autistic or not) and a set of input features for each test of each child:

- SCQ score.
- DwellTimeLeftEye (ms): time spent gazing on the left eye (human avatar).
- DwellTimeLeftEye_animal (ms): time spent gazing on the left eye (animal avatar).
- DwellTimeLeftFace (ms): time spent gazing on the left side of the face (human avatar).
- DwellTimeLeftFace_animal (ms): time spent gazing on the left side of the face (animal avatar).
- DwellTimeLeftSide (ms): time spent gazing on the left side of the screen (human avatar).
- DwellTimeLeftSide_animal (ms): time spent gazing on the left side of the screen (animal avatar).
- DwellTimeCongruent (ms): time spent gazing at the same object gazed upon by the avatar.
- TTFFLeftEye (ms): time it took for first fixation on left eye (human avatar).
- TTFFLeftEye_animal (ms): time it took for first fixation on left eye (animal avatar).
- TTFFLeftFace (ms): time it took for first fixation on left side of the face (human avatar).
- TTFFLeftFace_animal (ms): time it took for first fixation on left side of the face (animal avatar).
- TTFFLeftSide (ms): time it took for first fixation on the left side of the screen (human avatar).
- TTFFLeftSide_animal (ms): time it took for first fixation on the left side of the screen (animal avatar).
- TTFFCongruent (ms): milliseconds it took for first fixation on the same object gazed upon by the avatar.

### Machine learning-based classification

To identify the best algorithm for the classification problem at hand, we tested several popular machine learning algorithms, namely: logistic regression, support vector machines (SVMs), random forests (RF), and a custom-made long short-term memory (LSTM) neural network [19].

**Fig. 4:**
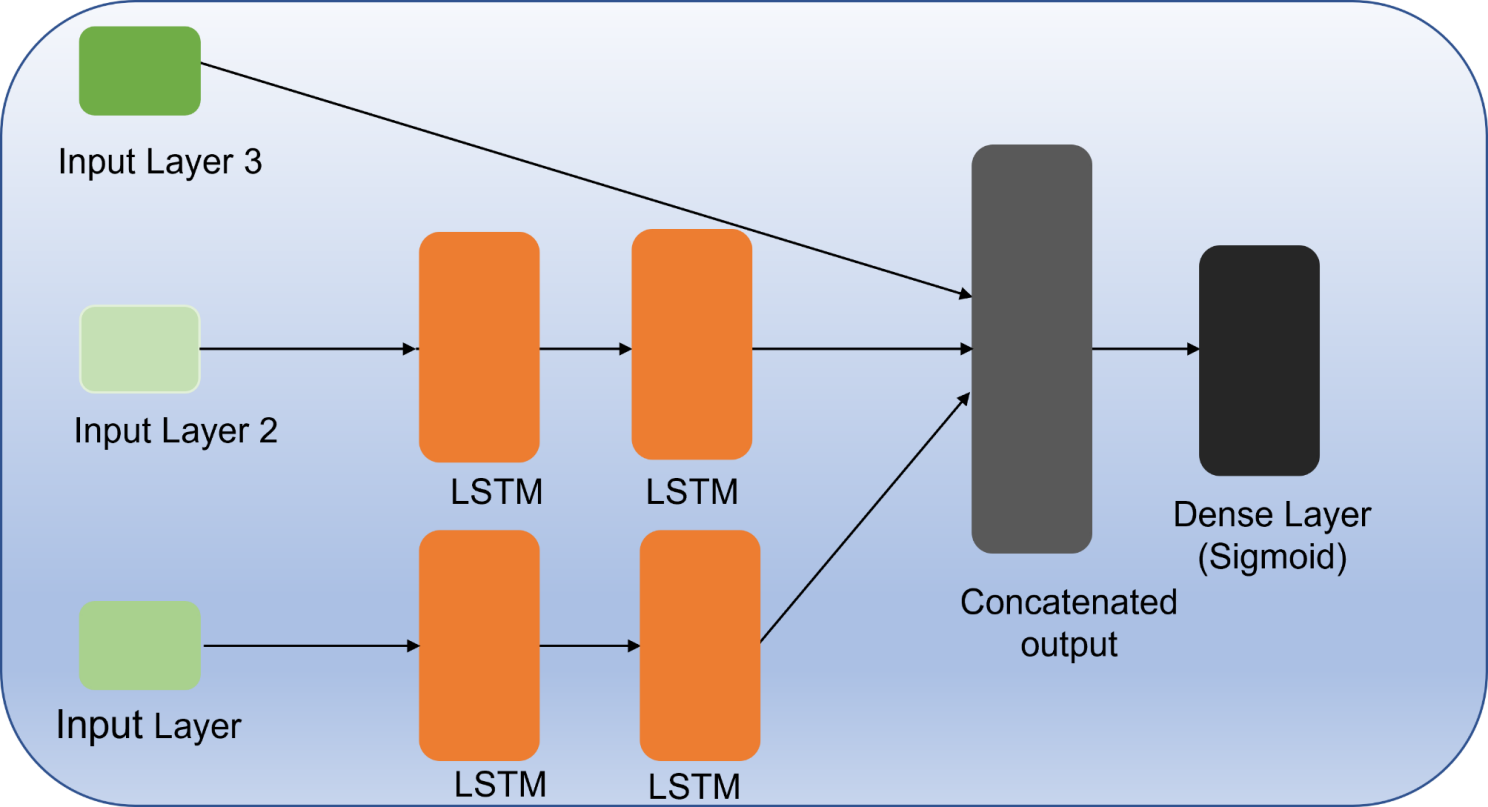
The architecture of the proposed LSTM network

Our LSTM model takes three inputs and predicts if the corresponding participant has ASD or not. Each input is fed to one of three branches of the model: one that takes as input the age and gender of the participant; one for the features relative to human avatars, organized as sequences of 20 values for each participant; and one for the features relative to animal avatars, organized as sequences of 5 values for each participant. The two sequence inputs are fed to two separate LSTM blocks, each consisting of one LSTM layer, which are particularly good at handling sequence data. The output of the two LSTM blocks is then concatenated (together with the scalar input) and fed to a dense layer, which performs the classification via a sigmoid activation function (Fig. 4).

As we had limited amounts of data (104 participants, each constituting a data point), we performed K-fold cross-validation. It involves splitting the dataset into K equally sized folds, training the model on K − 1 folds and evaluating its performance on the remaining fold. This process is repeated K times, with each fold serving as the validation set once, and the results are averaged to provide an overall estimate of the model’s performance. The technique helps to reduce overfitting and improve the generalization of the model. In this study we chose K = 5, although K = 10 is another equally valid and popular choice.

We compare the performance of all models using four metrics: accuracy, precision, recall, and F1. All traditional models were implemented using the Python library ‘sklearn’, whereas the LSTM model was implemented using the Python library ‘tensorflow’.

## Results

### Descriptive statistics

Table 1 below reports the average ± standard deviation values for all the features considered.

**Table 1:** Descriptive statistics of all the variables measured. TTF: Time to First Fixation (in ms)

Variables	ASD	Controls	
Prediction 1: Attention to the Eyes			
Dwell on eyes - Human	1424.93 ± 950.28	1578.64 ± 780.05	(t = 0.88(87.48), p = 0.38, d = -0.17)
Dwell on eyes - Animal	1210.68 ± 719.74	1456.34 ± 673	(t = 1.72(79.48), p = 0.09, d = -0.35)
Prediction 2: Left Visual Field Bias			
Left Face - humans	822.32 ± 416.11	980.26 ± 244	(t = 2.44(101.58), p = 0.02, d = -0.44)
Left Face - animals	1269.87 ± 830.61	1843.9 ± 722.72	(t = 3.66(83.65), p = 0.0004, d = -0.73)
Right Face - humans	780.76 ± 368.99	773.53 ± 254.29	(t = 0.12(96.92), p = 0.91, d = 0.022)
Right Face - animals	1256.62 ± 743.71	1458.08 ± 638	(t = 1.43(85.05), p = 0.16, d = 0.29)
Prediction 3 : Joint Attention			
Dwell - Congruent	975.77 ± 447.94	1421.17 ± 567.3	(t = 4.08(62.65), p = 0.0001, d = -0.91)
TTFF-Congruent	1097.49 ± 567.49	839.33 ± 424.72	(t = 2.57(91.97), p = 0.01, d = 0.5)
Dwell - Incongruent	769.83 ± 447.89	898.1 ± 323.58	(t = 1.63(91.02), p = 0.11, d = -0.32)
TTFF-Incongruent	1133.45 ± 577.64	1164.32 ± 485.03	(t = 0.28(83.85), p = 0.78, d = 0.057)

The groups showed no statistically significant difference in terms of dwell time on the eyes for human (t = 0.88(87.48), p = 0.38, d = -0.17) or animal (t = 1.72(79.48), p = 0.09, d = -0.35) faces, though for the animal stimuli there was a clear trend for the autism group to focus less on the eyes, with a moderate effect size.

Compared with the control group, the autism group had shorter dwell times on the left side of the face for both human (t = 2.44(101.58), p = 0.02, d = -0.44) and animal (t = 3.66(83.65), p = 0.0004, d = -0.73) faces. Differences in dwell time on the right side of the face were not significant across the groups for neither human (t = 0.12(96.92), p = 0.91, d = 0.022) nor animal (t = 1.43(85.05), p = 0.16, d = 0.29) faces. Thus, as expected, there was a reduced LVF bias in the autism group with a moderate to large effect size.

For the congruent (i.e., the object being looked at) object in the JA task, the autism group had longer time to first fixation (t = 2.57(91.97), p = 0.01, d = 0.5) and a shorter dwell time (t = 4.08(62.65), p = 0.0001, d = -0.91) than the control group. Effect sizes were large in both cases. There were no significant differences in dwell time (t = 1.63(91.02), p = 0.11, d = -0.32) or time to first fixation (t = 0.28(83.85), p = 0.78, d = 0.057) on JA incongruent objects. Thus, as expected, the autism group showed a reduction in reflexive joint attention.

### Model results

The performance of all models is reported in Table 2, where the values reported indicate mean ± confidence intervals, averaged over the K folds. As can be seen, the random forest model is the best performing one under all metrics.

**Table 2:** Evaluation metrics for the models considered

Model	Accuracy	Precision	Recall	F1
Logistic regression	0.59 ± 0.13	0.66 ± 0.17	0.65 ± 0.16	0.65 ± 0.15
SVM	0.61 ± 0.09	0.66 ± 0.15	0.80 ± 0.12	0.71 ± 0.09
Random forest	**0.76 ± 0.08**	**0.78 ± 0.13**	**0.84 ± 0.07**	**0.80 ± 0.09**
LSTM	0.75 ± 0.15	0.60 ± 0.32	0.80 ± 0.39	0.68 ± 0.34

## Discussion

In this study, we first examined whether Saudi children with and without autism could be differentiated based on three types of eye tracking metrics: dwell time on the eyes, dwell time on the left side of faces, and joint attention. The results revealed that the autism group displayed deficits in reflexive JA and had a weaker LVF bias during face perception compared with the controls, showing these stimuli are useful for characterizing deficits in social processing and communication. However, in terms of time spent looking in the eyes, although the ASD group spent on average less time doing so, the statistical comparison did not reach significance. Overall, the results show that the combination of these metrics was sensible for accurate autism identification at the individual level in our study cohort, making it a candidate biomarker worthy of further study.

We then tested a series of machine learning algorithms, to understand if they could learn to classify ASD by analyzing eye tracking data. Of the models tested, the random forest model proved to be the best across all metrics. Interestingly, the LSTM model, which is by far the most complex and novel of the ones tested, performed quite poorly, surpassing only logistic regression (the most basic algorithm) in terms of F1 score [3]. We attribute this to the fact that more complex models tend to perform worse than simpler ones in data-scarce applications, such as the one at hand.

The performance of the random forest model suggests it might be a viable screening tool for autism in Saudi children. However, considering the context of its usage is crucial. Although the assessment may have some usefulness in suspected at-risk populations, the prevalence differences between autism and non-autism suggest that the rates of false positives would be unacceptably high for general screening purposes. Further investigation will be required to delve into these subjects. It is worth noting that while we drew on previous autism research to shape our study, it was not immediately apparent that the metrics we considered would effectively differentiate the groups or accurately classify individual children in our specific target population of Saudi children. There is a lack of research on this topic for populations in Arabian countries, as well as outside of Europe and the US.

While our study represents an important initial stride, additional research is necessary to assess the extent to which we can distinguish autism from other clinical categories, such as non-autistic children with intellectual disability or ADHD, using these eye tracking-based measures of social processing [20]. In other words, we need to determine the accuracy of this battery for differential diagnosis. A second limitation of our study lies in its utilization of a small sample size. Although we took precautions to mitigate overfitting by dividing the sample into training and validation sets, the test set consisted of only a small group. Moreover, small sample sizes are more susceptible to the influence of random factors, which prevented us from exploring the specific differences between those misclassified (few false negatives and more common false positives) and the rest of the participants. It should be noted that these biomarkers could potentially be employed for stratification within the ASD population, but this would necessitate larger sample sizes. Additionally, our knowledge about the NT group included in our study is relatively limited. For example, we lack information on the extent to which this group is matched with the ASD group in terms of IQ and other measures. A final limitation stems from the COVID restrictions and mask policies, which may have distracted certain participants and altered their typical social interaction behaviors. Despite acknowledging these challenges and limitations, we consider the results of the current study to be promising, particularly since we were able to demonstrate these patterns of difficulty in the ASD group within an underexplored geographical context. Ultimately, a reliable, accurate, and user-friendly autism marker could significantly aid diagnostic decision-making and prognostic predictions.

## Conclusions

The aim of this study was to develop a machine learning algorithm capable of classifying Saudi children with or without autism by analyzing eye-tracking data. We show that the random forest model achieves higher performance than other traditional machine learning algorithms and can distinguish between Saudi children with and without autism, though specificity limitations suggest that further research and development are required.

## Data Availability

The datasets used and analyzed during the current study are available from the corresponding author upon reasonable request and fulfilment of the organization requirements.

## Abbreviations

ADHD: Attention deficit hyperactivity disorder
ADOS: Autism Diagnostic Observation Schedule
AOI: Areas of interest
ASD: Autism Spectrum Disorder
DSM-5: Diagnostic and Statistical Manual of Mental Disorders, Fifth Edition
JA: Joint attention
LSTM: Long Short-Term Memory
LVF: Left visual field bias
ML: Machine learning
NINDS: National Institute of Neurological Disorders and Stroke
SCQ: Social Communication Questionnaire
TTFF: Time to first fixation
